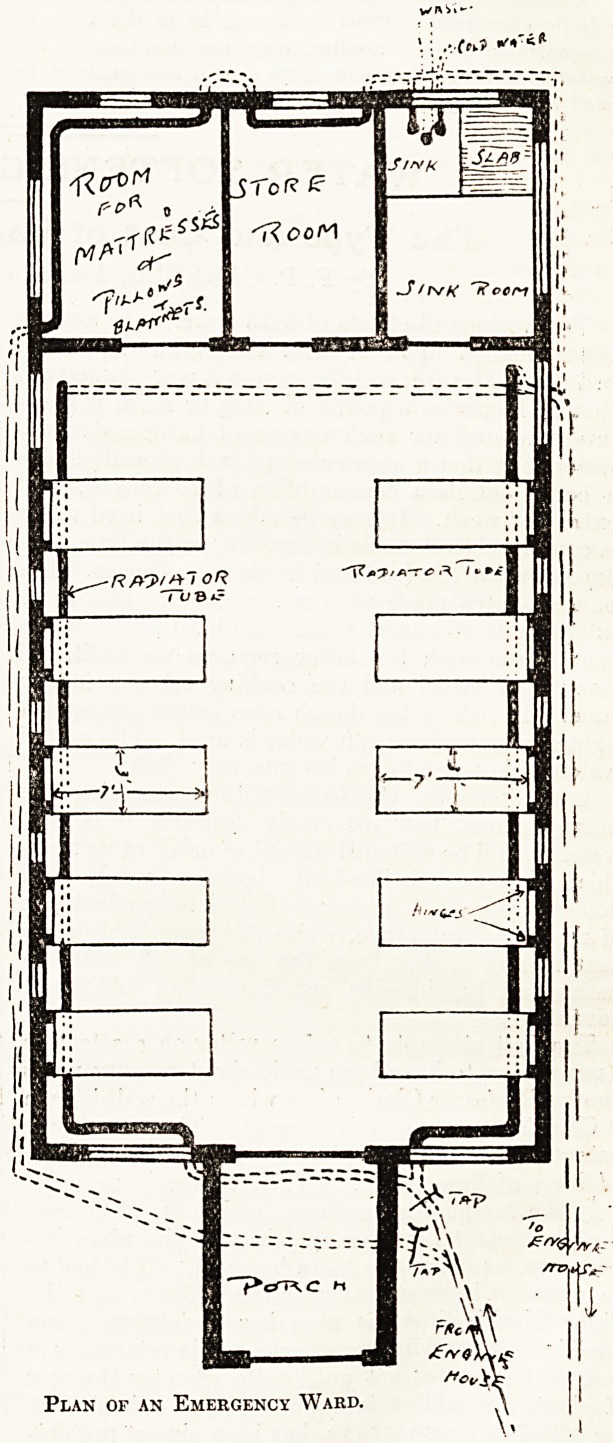# A Design for Emergency Hospitals in Industrial Districts

**Published:** 1912-06-22

**Authors:** 


### HOSPITAL ARCHITECTURE AND CONSTRUCTION.

**[Communications on this subject should be marked "Architecture" in the left-hand top corner of the envelope.]**

### A Design for Emergency Hospitals in Industrial Districts.

### THE NEED FOR COMPULSORY ACCOMMODATION.

To anyone in the position of house surgeon to a hospital serving an industrial district or a large mining centre, or to a surgeon acting in one of these districts, it is a matter of the greatest surprise that a worker who may meet with any severe accident is compelled to run tremendous risks before he can receive succour.

For instance, a man gets badly burnt by an explosion in a coal mine, or is crushed by a falling heavy body. The shock consequent on such an accident is very severe, and is generally the direct cause of an early fatal termination. Putting all else aside, it would be thought that employers would try to safeguard their own interests; or that the insurance companies, which now take the burden of compensation on their own shoulders, would lessen the premium to a works or mine that safeguarded their risks to a considerable extent.

The writer has seen many patients who, if they had been treated on the spot for shock, combined with very rough first-aid to the actual injury itself, would now have been living and earning good wages; but the delay in getting some kind of conveyance, and then the further exposure (sometimes on an open trolley) during a long drive into the nearest town with an infirmary, turned the scale. The shock due to the accident, very severe in itself but not necessarily fatal, has been augmented to such a degree that the last hope of recovery has been ruthlessly taken away for no reason at all beyond ignorance and carelessness on the part of those who profess to care for the welfare of their employees.

If a small hospital ward of the type shown in ground plan in the accompanying figure were made a compulsory part of any works establishment or pit-head buildings, then it would be possible to render efficient first-aid to any sufferer and to treat the shock by efficient means. The mortality from accidents would thus be appreciably lessened.

Every severe accident entails a very great degree of shock. The shock requires to be treated by absolute rest, by applications of heat to the body from head to foot, by giving stimulants such as hot drinks, and by a general atmosphere of at least 70° to 80° F. in the surrounding air. Such conditions would be available within fifteen minutes if the following scheme were carried out in its entirety. The cost would be less than £200, as the building need be only of match-boarding and corrugated iron. The hot-water supply to the large tubes running round the walls and to the sink-room would come straight from the main engine-house. The tap controlling the main supply to these tubes would be at the hospital door. A separate pipe always at work would circulate the water, day and night, through the rooms set apart for mattresses and blankets, and for drugs and dressings. The rest of the room would be devoted to bed space, although in cases of severe accidents it would be necessary to use all the floor space. The beds would consist of planks of wood seven feet by three feet, and two inches thick. They would be fastened to the wall by strong hinges at a height of two feet from the ground. The other end would be supported by folding legs, perfectly safe and rigid when in use. There would be no boards at the head or foot, and so, when not required, the beds could be pushed up against the wall and held in this vertical position by clips. Some small shelves could be fitted on the underside of the plank which would be out of the way when the bed was in use, yet could be made of great service

Plan of an Emergency Ward.

where space is valuable when the beds were all pushed back.

Anything put into the room, when not needed for its special purpose, should be very easily portable so that the room could be cleared entirely at ten minutes' notice.

On word being received of a severe accident the room would be cleared and as many beds as necessary put down. Then the warm mattresses and pillows would be got out of their store-room and placed on the beds. Next the hot-water bottles in the middle room would be filled from the hot-water supply in the sink-room. The water to the main radiators should have been turned on before anything else was begun.

A stock of Bovril and soup should be found in the middle store-room. The soup should be in the form of concentrated liquid, needing only the addition of hot water to make it. Feeding cups should also be there, as well as dressings and drugs.

If a nurse were employed by the pit or works club to attend to the workmen and their families as ordinary routine work, and to live as near to the works as possible, she would naturally take charge of this emergency hospital and render such first-aid as could be done till the doctor arrived, for he is always sent for on such occasions. The sufferers would be taken to the larger and better equipped local infirmary as soon as possible after the abatement of the shock, as it is not intended to nurse them to recovery in this small hospital. It is only designed to be used as a means to combat shock, and by so doing to take away the most potent cause of death after otherwise only moderately severe accidents.

## Figures and Tables

**Figure f1:**